# A New Cell-Selective Three-Dimensional Microincubator Based on Silicon Photonic Crystals

**DOI:** 10.1371/journal.pone.0048556

**Published:** 2012-11-06

**Authors:** Francesca Carpignano, Gloria Silva, Salvatore Surdo, Valentina Leva, Alessandra Montecucco, Francesca Aredia, Anna Ivana Scovassi, Sabina Merlo, Giuseppe Barillaro, Giuliano Mazzini

**Affiliations:** 1 Dipartimento di Ingegneria Industriale e dell’Informazione, Università di Pavia, Pavia, Italy; 2 Dipartimento di Ingegneria dell’Informazione, Elettronica, Informatica, Telecomunicazioni, Università di Pisa, Pisa, Italy; 3 IGM-CNR, Pavia, Italy; 4 Dipartimento di Biologia e Biotecnologie “L. Spallanzani”, Università di Pavia, Pavia, Italy; University of Cincinnati, United States of America

## Abstract

In this work, we show that vertical, high aspect-ratio (HAR) photonic crystals (PhCs), consisting of periodic arrays of 5 µm wide gaps with depth of 50 µm separated by 3 µm thick silicon walls, fabricated by electrochemical micromachining, can be used as three-dimensional microincubators, allowing cell lines to be selectively grown into the gaps. Silicon micromachined dice incorporating regions with different surface profiles, namely flat silicon and deeply etched PhC, were used as microincubators for culturing adherent cell lines with different morphology and adhesion properties. We extensively investigated and compared the proliferative behavior on HAR PhCs of eight human cell models, with different origins, such as the epithelial (SW613-B3; HeLa; SW480; HCT116; HT29) and the mesenchymal (MRC-5V1; CF; HT1080). We also verified the contribution of cell sedimentation into the silicon gaps. Fluorescence microscopy analysis highlights that only cell lines that exhibit, in the tested culture condition, the behavior typical of the mesenchymal phenotype are able to penetrate into the gaps of the PhC, extending their body deeply in the narrow gaps between adjacent silicon walls, and to grow adherent to the vertical surfaces of silicon. Results reported in this work, confirmed in various experiments, strongly support our statement that such three-dimensional microstructures have selection capabilities with regard to the cell lines that can actively populate the narrow gaps. Cells with a mesenchymal phenotype could be exploited in the next future as bioreceptors, in combination with HAR PhC optical transducers, e.g., for label-free optical detection of cellular activities involving changes in cell adhesion and/or morphology (e.g., apoptosis) in a three-dimensional microenvironment.

## Introduction

The rapid progress in the development of new micro- and nano-technologies together with the improvements in cell culture has led to the development of cell biosensors for clinical diagnostics, drug discovery, and detection of toxic agents or food research [1]–[4], with consequent direct benefits for human health and undoubted advantages in terms of industrial efficiency and animal welfare. These sensors use living cells as bioreceptors and allow cell morpho-functional changes and/or detachment, induced by exposure to environmental perturbations, to be monitored by a suitable transduction method (i.e., optical, electrical). Cells, as bioreceptors, probe the presence/action of a bioactive agent meanwhile the transducer translates the resulting cellular response into an electrical or optical signal that can be processed and analyzed, possibly in a non-invasive manner.

Although cells typically reside *in vivo* in a three-dimensional (3-D) environment, most of what is known about cells has been derived from cultures performed on flat surfaces, such as plastic Petri dishes, flasks or glass slides [5]. Therefore, there is an increasing interest in investigating cell cultures on 3-D matrices, also known as scaffolds, since these structures can have major effects on cell behavior [6], [7] with regard to adhesion [8], [9], proliferation, differentiation and, also, apoptosis [10], [11]. Conventional two-dimensional cell cultures do not reproduce the *in vivo* tissue architecture, and do not, for example, forecast organ-specific toxicity [12]. On the other hand, 3-D cultures emulate the biochemistry and mechanics of the microenvironment in tissues more closely [13]. The use of *in vitro* three-dimensional cell cultures could reduce the high cost of animal experiments, that are not always predictive of the human response, e.g., in the case of toxicity testing [12]. Several scientists have demonstrated that cell behavior, but also the differentiation of stem cells [14], are affected by the topography of the underlying surface [15]. *In vitro* models have great potential for *ex vivo* culture of primary cells, particularly human stem cells. The majority of stem cell research has been performed in animal models because of their complex natural microenvironment. A stem cell environment, faithfully reconstructed *in vitro*, could provide a deep understanding of human stem cell biology and elevate its clinical potentials [13] for regenerative medicine [16]. Therefore, 3-D cell cultures represent a potential bridge to cover the gap between animal models and human studies.

**Figure 1:**
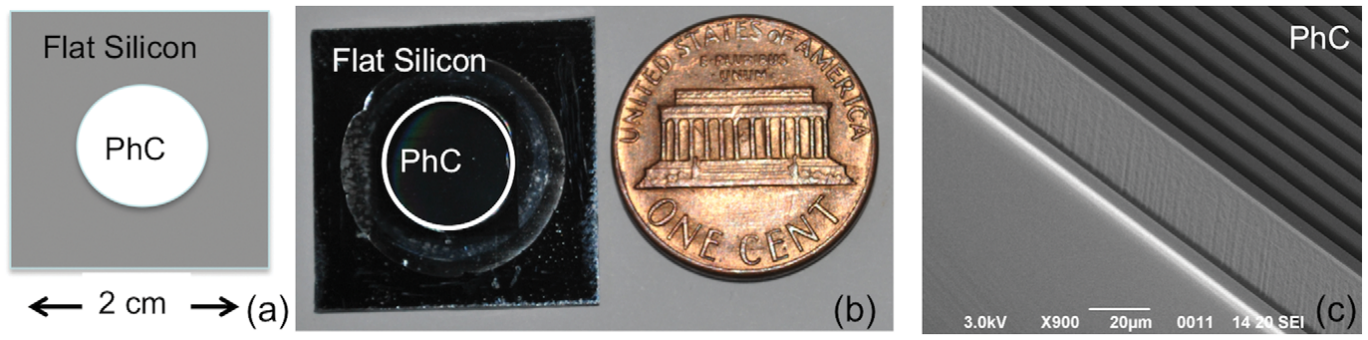
Images of silicon devices. a: Schematic drawing of the different regions on a silicon chip. b: Photo of the device together with a reference size. c: Scanning Electron Microscopy image of the three-dimensional silicon microstructure.

Usually, the considered matrices, or scaffolds, are porous substrates, for example hydrogels [17], that can support cell growth, organization, and differentiation on or within their structure. More recently, cell growth, attachment and response were investigated on 3-D isotropic silicon microstructures, fabricated by reactive ion etching, consisting in microchambers with diameter in the range 150–170 µm and depth 60–70 µm [18]–[21].

We have recently proposed the use of silicon devices based on a well-ordered material as a three-dimensional supporting matrix for biological nanostructures [22] and for optofluidic applications [23]. In this paper, a cell-selective silicon microincubator, that incorporates a vertical, high aspect-ratio (HAR) silicon photonic crystal (PhC) as core element, is successfully demonstrated for performing cell cultures in a 3-D microenvironment. HAR PhCs consist of periodic arrays of parallel ≈ 3 µm-thick silicon walls separated by ≈ 5 µm-wide, 50 µm-deep air gaps, fabricated by electrochemical micromachining (ECM) of <100>-oriented silicon wafers [24], [25]. PhCs are artificial materials characterized by the presence of photonic bandgaps, i.e., wavelength intervals in which the propagation of the electromagnetic field inside the material, in our case in direction orthogonal to the silicon walls, is prohibited [26].

**Figure 2:**
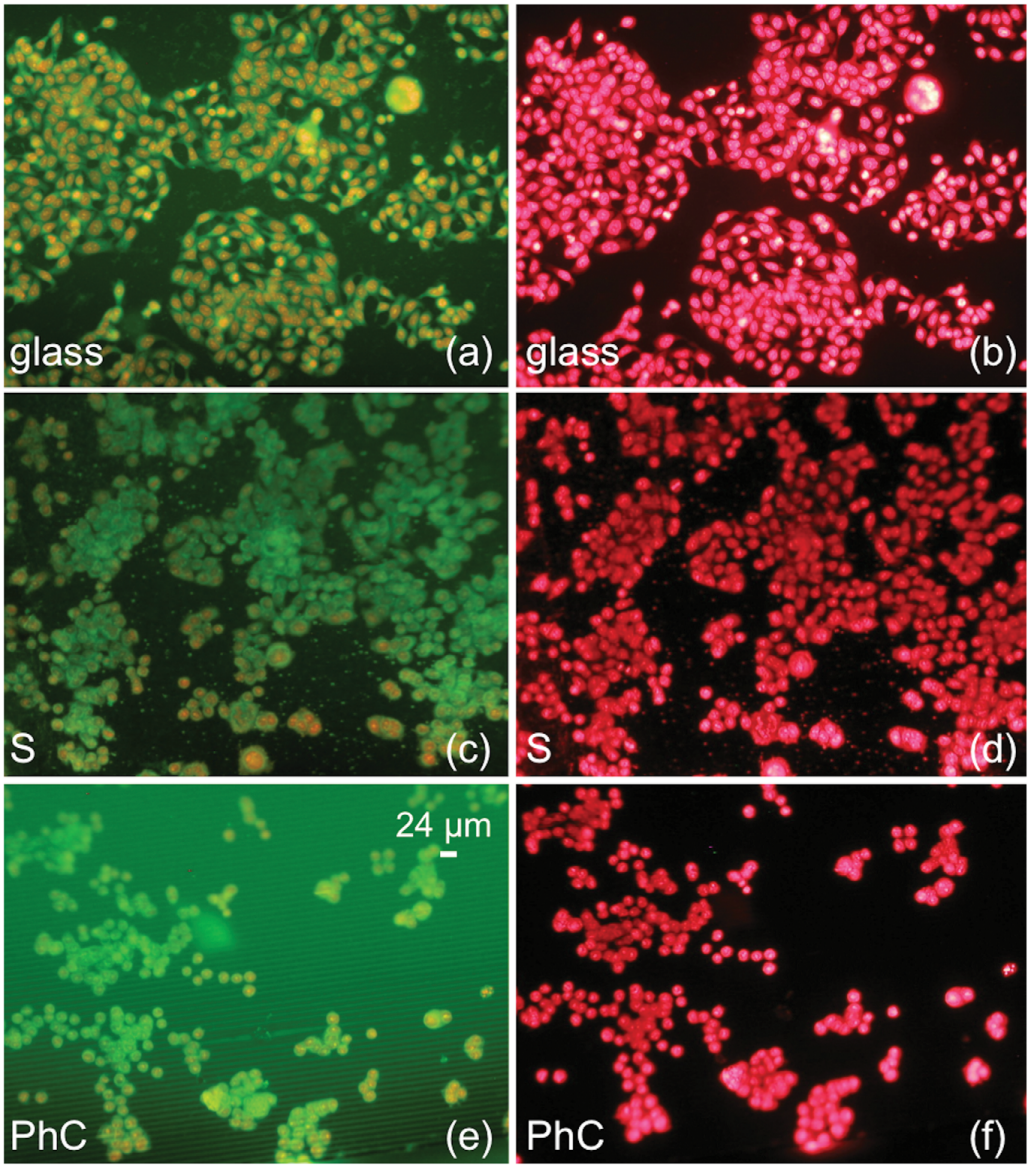
Fluorescence images relative to HT29 cells. a–b: Cell morphology on a glass slide (glass). c–d: Cell morphology in a flat silicon region (S). e–f: Cell morphology in the Photonic Crystal (PhC). Cells are labeled with (a, c, e) green-FITC and red-PI; (b, d, f) only red-PI.

The definition of interaction effects between PhCs and cell cultures represents a first significant step toward the fabrication of a new-concept cell-based optical biosensor, in which cells grow and proliferate into HAR PhC transducers, for direct label-free optical monitoring of cellular activities. In fact, changes in cell morphology and distribution inside the gaps could strongly affect the optical properties of the photonic bandgap material. These silicon micromachined structures could, thus, have a potential as new tools for studying the biological properties of rare cell subpopulation having a relevant clinical interest in tumor metastasis, such as cancer stem cells.

Although preliminary data relative to cell growth on similar silicon structures were previously reported [27], we have more extensively investigated and compared the proliferative behavior on HAR PhCs of eight cell lines, mainly belonging to two different embryonic-lines, namely the epithelial (SW613-B3; HeLa; SW480; HCT116; HT29) and the mesenchymal (MRC-5V1; CF; HT1080). Cell cultures on micromachined silicon chips were analyzed by fluorescence microscopy, after labeling with Propidium Iodide and Fluorescein Isothiocyanate [28]. New experimental results, reported in this work, strongly support our statement that cells with a mesenchymal behavior are better prone to actively colonize the gaps, adherent to the inner vertical surfaces of the silicon walls. This feature provides the evidence that these HAR micromachined structures, as microincubators, exhibit a sort of cell selectivity. For comparison, contributions of cell sedimentation and/or simple “physical trapping” of cells inside the silicon gaps were also investigated. Cells with a mesenchymal phenotype could actually be exploited in the near future as bioreceptors, in combination with HAR PhCs that will then play the role of 3-D microincubators involving small amounts of cells and reagents, potentially working also as optical transducers.

**Figure 3:**
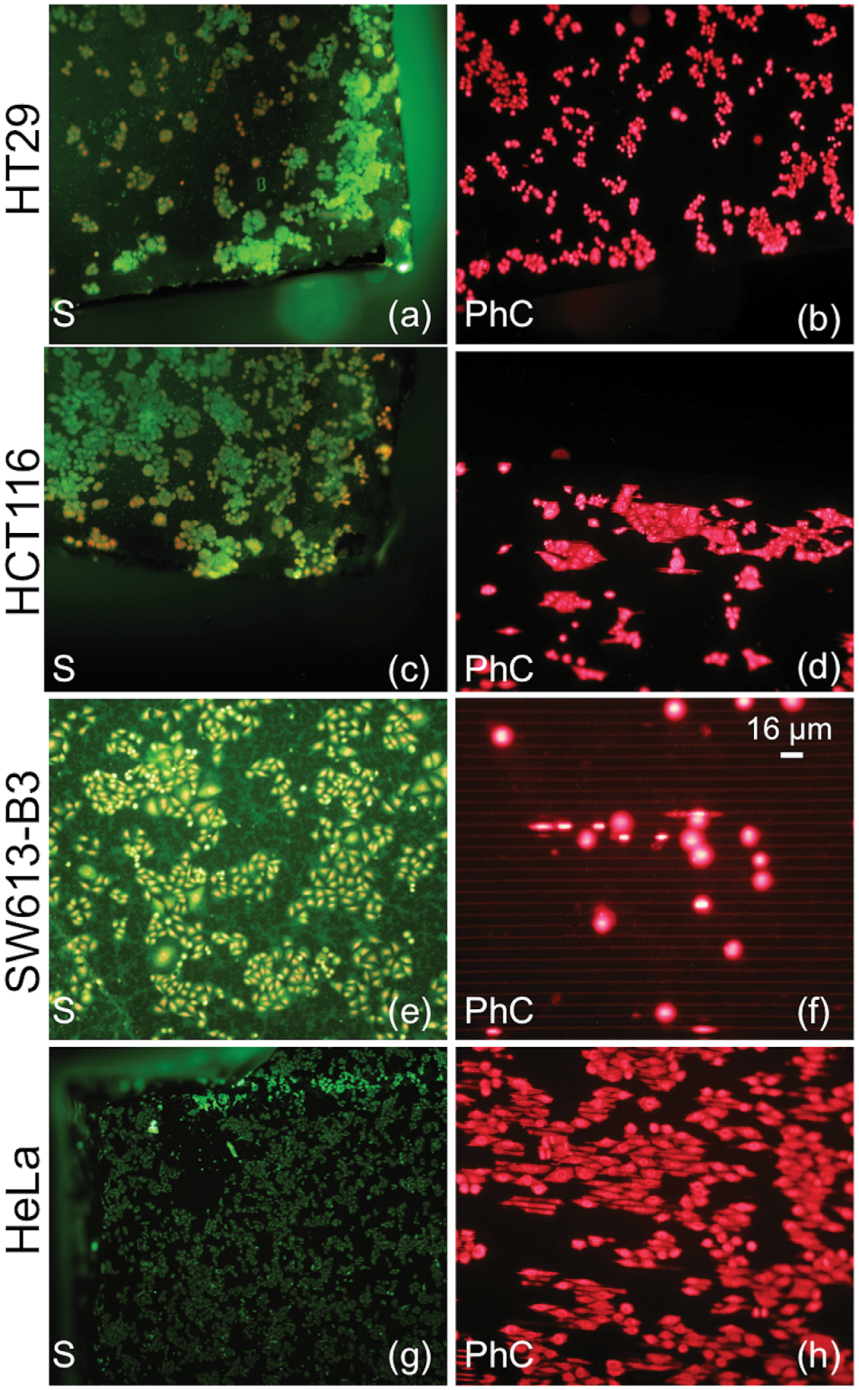
Comparison between fluorescence images relative to different epithelial cell lines. a–b: HT29. c–d: HCT116. e–f: SW613-B3. g–h: HeLa. (a, c, e, g) Cell morphology in a flat silicon region (S); (b, d, f, h) Cell morphology in the Photonic Crystals (PhC). Cells are labeled with (a, c, e, g) green-FITC and red-PI; (b, d, f, h) only red-PI. In (b,d,f,h), photos in the right column, the majority of the nuclei have a round shape, typical of cells staying on top of the walls.

**Figure 4:**
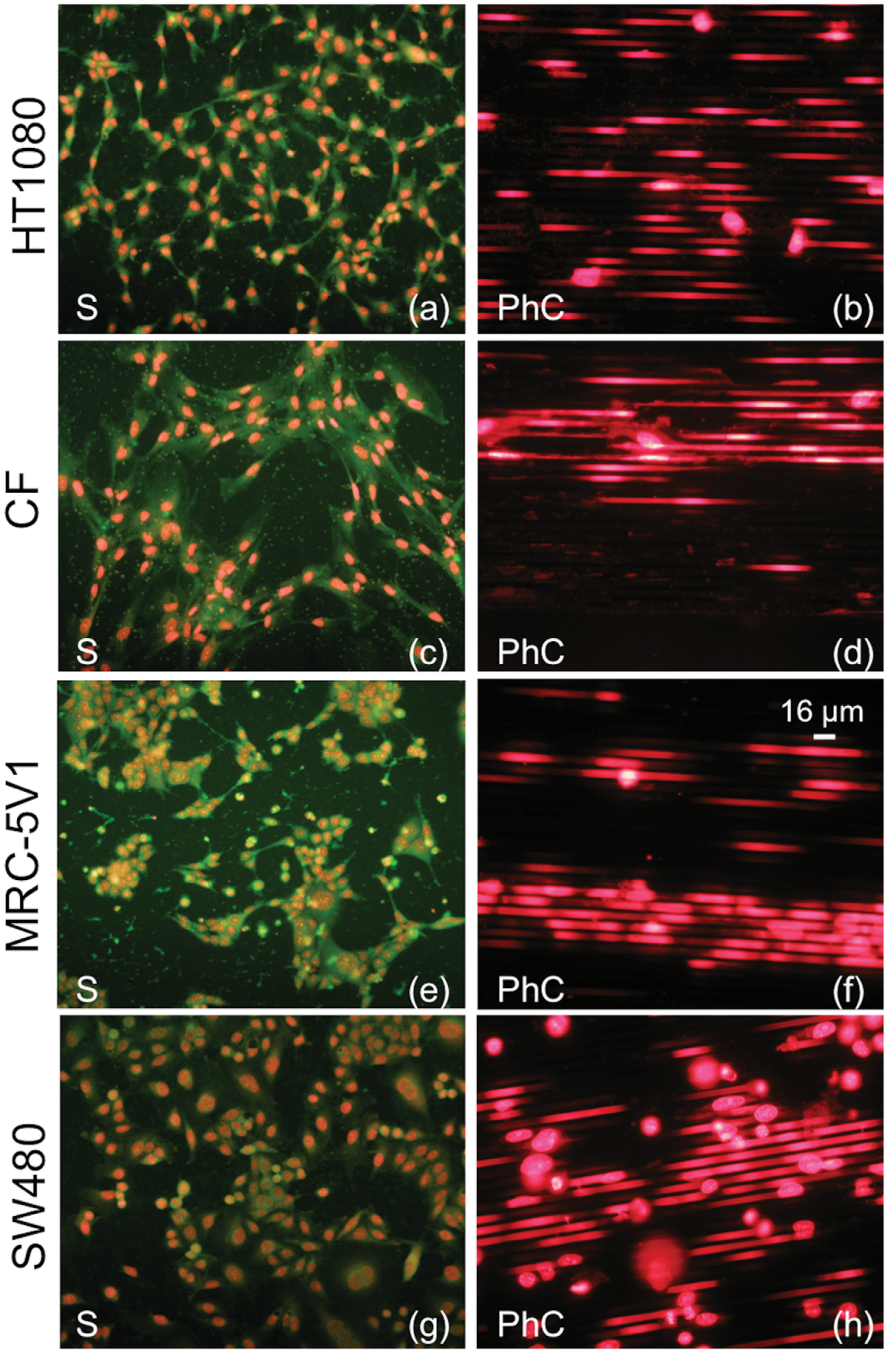
Comparison between fluorescence images relative to cell lines exhibiting mesenchymal behavior. a–b: HT1080. c–d: CF. e–f: MRC-5V1. g–h: SW480. (a, c, e, g) Cell morphology in a flat silicon region (S); (b, d, f, h) Cell morphology in the Photonic Crystal (PhC). Cells are labeled with (a, c, e, g) green-FITC and red-PI; (b, d, f, h) only red-PI. In (b,d,f,h), photos in the right column, the majority of the nuclei have an elongated shape, typical of cells inside the gaps.

## Materials and Methods

### Silicon Microstructure Fabrication

Silicon devices were fabricated by means of the electrochemical micromachining technology (ECM) and details are reported in [24]. At the end of the fabrication process, each silicon chip contains the electrochemically etched, central region (with circular shape and area of 0.64 cm^2^) incorporating the HAR PhC, surrounded by flat silicon. In this work, we used photonic crystals with long walls (0.5–1 cm) as well as with short (400 µm) walls, characterized by increased rigidity and robustness. Some silicon micromachined dice were trimmed to fit in 12-well plates.

**Figure 5:**
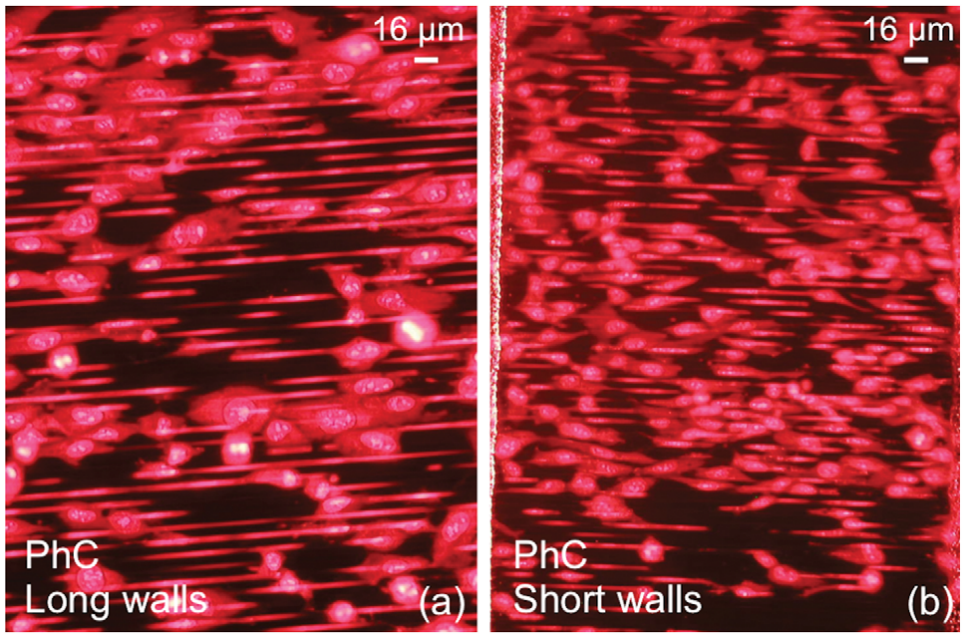
Comparison between fluorescence images relative to HT1080 cells in Photonic Crystals. a: PhC with long walls. b: PhC with short walls. No significant difference is observed between (a) and (b) since the length of the walls does not affect the cell behavior.

**Figure 6:**
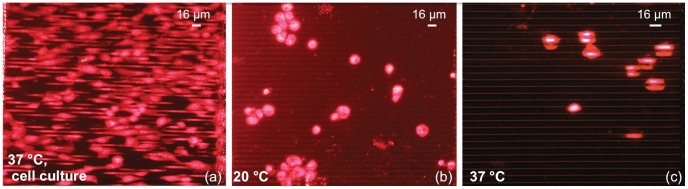
Comparison between fluorescence images relative to HT1080 cell culture in different conditions on PhC with short walls. a: Standard culture. b: Cell deposition at room temperature (∼20°C). c: Cell deposition at 37°C.

### Cell Lines and Reagents

SV40-transformed fibroblasts MRC-5V1 were purchased from European Collection of Cell Cultures (No. 85042501), SW480 colon adenocarcinoma and HeLa cells were purchased from American Type Culture Collection (ATCC No. CCL-228 and CCL-2.2, respectively).

HCT116 and HT29 cells were obtained respectively from C.R. Boland (University of California, La Jolla, USA) [29] and R. Supino (Istituto Nazionale Tumori, Milano, Italy; [30]). The fibrosarcoma HT1080 cell line was a gift of M. Ciomei (Nerviano Medical Sciences, Italy) [31]. SW613-B3 cells were isolated and provided by O. Brison (IGR Villejuif, France) [32]. Embryonic CF fibroblasts were established *in vitro* and kindly provided by T. Nardo (IGM-CNR, Pavia, Italy).

All the examined human cell lines were grown as monolayers. SW613-B3 cells (from colon carcinoma) and fibrosarcoma HT1080 cells were grown in complete DMEM supplemented with 10% Fetal Bovine Serum (FBS), glutamine (4 mM), Na/pyruvate (2 mM), penicillin (100 U/ml) and streptomycin (0.1 mg/ml). SV40-transformed fibroblasts MRC-5V1, HeLa cells and CF embryonic fibroblasts were grown in complete DMEM supplemented with 10% FBS, glutamine (4 mM), and gentamicin (50 µg/ml). SW480 colon adenocarcinoma cells were grown in complete RPMI supplemented with 10% FBS, glutamine (2 mM), Hepes (25 mM) and gentamicin (80 µg/ml). Colorectal carcinoma HCT116 cells were grown in complete RPMI supplemented with 10% FBS, glutamine (4 mM), Na/pyruvate (2 mM), penicillin (100 U/ml) and streptomycin (0.1 mg/ml). Colon adenocarcinoma HT29 cells were grown in complete McCoy supplemented with 10% FBS, glutamine (4 mM), Na/pyruvate (2 mM), penicillin (100 U/ml) and streptomycin (0.1 mg/ml). All reagents were from Celbio, Lonza and Sigma, Italy.

### Cell Culture in Standard Condition (Before Seeding on Silicon Devices)

All cells were normally grown at 37°C in a humidified atmosphere containing 5% CO_2_. Growth conditions were monitored daily by direct observation with an inverted microscope and, when confluence was reached, they were trypsinized as previously described in [33]. Some cells were used in subsequent experiments of seeding and growth in contact with silicon whereas the cell line was maintained by re-seeding the remaining cells in plastic plates or flasks.

### Cell Seeding on Silicon

To define the best culture conditions on silicon and to define the optimal density of cells inside the grooves, we used different concentrations of cells in the medium and increasing culture times. Culture experiments were performed on as-cut or trimmed silicon dice fitting, respectively, in 6- or 12-well plates. Seeding was carried on in two distinct ways: by depositing a drop of medium containing cells over the silicon dice and adding 2 ml of medium only 3 h later, or by directly seeding in the well, hosting the silicon dice, 2 ml of medium containing 3×10^5^ cells.

### Cell Culture on Silicon Samples

Cell culture experiments were repeated several times, as previously described: the 24 h incubation of cells directly into the well ensured a sufficient cell density. Sterilized micromachined silicon devices fitting in 12-well plates were incubated as usual at 37°C in a humidified atmosphere containing 5% CO_2_ for 24 h, before fixing.

### Influence of “Cell Sedimentation”

To evaluate the contribution of bare “cell sedimentation” on silicon device colonization, we performed the following tests:

- a drop of cell suspension in isotonic medium (Phosphate Buffer Solution, PBS) without nutrients was placed on silicon, at the same cell concentration as previously described, and maintained at 37°C for 2 h;
- a drop of cell suspension was placed on silicon as in (a) but maintained at room temperature (at 20°C) for 2 h.

These experiments were designed to verify the eventual influence of spontaneous sedimentation, on cell penetration, inside the silicon grooves also as function of viscosity and/or temperature of the medium. Also these samples were, then, fixed and labelled as described in the next paragraph.

### Fluorescence Microscopy Analysis

At the end of the incubation time, after the medium was removed, silicon dice were gently washed (twice) with PBS (0.1 M), finally replaced with cold (−20°C) 70% ethanol (2 ml) for cell fixation. The silicon dice were stored after cell fixation at 4°C. In order to evaluate the cell density and morphology, the samples were stained with a combination of two fluorochromes for specific labeling of the nuclear DNA (Propidium Iodide (red-PI)) and the cytoplasm components (Fluorescein Isothiocyanate (green-FITC)). A mixture of dyes, 1 µg/ml PI and 0.1 µg/ml FITC, in PBS applied for 30 min was used for cell labeling, suitable for fluorescence observation. In order to prevent sample drying during microscopy observation, the silicon dice were washed in PBS and transferred on glass slides and covered with cover slips.

All samples were observed with an Olympus BX51 microscope with standard fluorescence equipment (HBO100/2 lamp). Excitation and emission filters combinations for single color (red-PI) as well as for simultaneous dual color (green-FITC and red-PI) imaging were applied. Blue excitation for dual color green-FITC/red-PI was performed with a band pass (BP 450–480 nm) excitation filter through a dichroic mirror DM500 combined with a LP 515 nm as barrier filter. Green excitation for single color red-PI was performed with a BP 530–560 nm and a dichroic mirror DM590 combined with a LP 620 as barrier filter. Fluorescence microphotographs with different magnifications (Olympus UPlanFl Objectives 20× NA = 0.50, 40× NA = 0.75) were taken using an Olympus Camedia C-4040 digital camera.

## Results

Examples of silicon devices, obtained after the technological process described in the Materials and Methods, are illustrated in Figure 1. Figures 1a and 1b show, respectively, a scheme and a photo of HAR PhC with a dimensional reference whereas Figure 1c reports a Scanning Electron Microscope (SEM) picture of HAR PhCs.

As a result of the various experiments of cell seeding on silicon, we verified that seeding on silicon dice fitting in a 12-well plates allowed the best density to be reached by minimizing the number of cells that remain attached to the surrounding plastic surface of the well, instead of silicon. Moreover, the 24 h culture time ensured a sufficient cell density with a few cells overlapping and without the need to replace the culture medium.

To establish a more general finding relative to the capability of cells to actively colonize the deep gaps of silicon HAR PhCs, we investigated the behavior of eight cell lines with different morphology and adhesion properties. The MRC-5V1, CF and HT1080 lines are cells with a mesenchymal phenotype characterized by a spindle-shaped morphology with migratory protrusions and capability [34]. These cells are able to migrate as individual cells through the extracellular matrix to which they adhere, and rarely establish direct contact with neighboring cells. On the other hand, SW613-B3, HeLa, HCT116 and HT29 epithelial cells tend to form a sheet or layers of cells that are tightly connected. Under normal conditions epithelial cells are unable to establish strong interactions with the underlying extracellular matrix. Finally, we exploited primary adenocarcinoma SW480 cells that, despite their epithelial origin, undergo the epithelial to mesenchymal transition (EMT) when grown at low density [35], and also in our experimental conditions they exhibited a mesenchymal behavior.

As reported in Figure 2 for HT29 cell culture, as a representative example, the comparison among cells grown on flat silicon substrates (S), Figures 2c–d, or on conventional glass slides (glass), Figures 2a–b, showed no appreciable differences in cell density and/or morphology. These results are in agreement with the fact that the physico-chemical properties of a silicon surface are very similar to those of glass slides. Cells were cultured also on silicon dice incorporating the HAR PhC to investigate the effect of surface texture, as reported in Figures 2e–f for the same line. The image shows that the morphology of cells grown on deeply etched regions resembles the morphology exhibited by cells on flat silicon and glass slides. The use of dice that incorporate two different regions allows a direct comparison of the cell behavior on standard flat silicon and PhC to be performed, thus eliminating spurious effects due to the typical biological variability; in our experiments cells grow on flat silicon and PhC at the same time, in the same environmental conditions. Fluorescence microscopy images of cells on flat silicon surfaces reveal that all investigated cell lines exhibit their typical morphology and demonstrate their ability to stretch the cytoplasm to form bridges towards other nearby cells, or simply to explore specific anchoring of the surrounding space. On the other hand, the behavior of the tested cell lines is quite different in the region with deep walls: cells with a mesenchymal phenotype grow preferentially inside the gaps, linked to the silicon walls, while epithelial cells remain mainly on the top of the silicon walls where they tend to form colonies. The cell behavior is shown in Figures 3 and 4 for epithelial and mesenchymal phenotypes, respectively, and it is strongly related to the three-dimensional microenvironment. For a better understanding of the different behavior of epithelial and mesenchymal cells, a direct comparison between the photos shown in the right columns of Figure 3 and 4 should be performed, taking into account that the shape of the nuclei is correlated to the cell position. The majority of the epithelial cells grown on PhCs (Figure 3b,d,f,h) maintains a round-shaped nucleus whereas most of the cell with mesenchymal behavior (Figure 4b,d,f,h) exhibit nuclei with a stretched shape. Round nuclei represent cells on top of the walls whereas elongated nuclei are typical of cells inside the gaps. Figure S1 shows in more details how the position of the cell is correlated to the shape of the nuclei. Cells on top of the silicon walls have a limited ability to proliferate and divide on a flat horizontal surface, since it is only a few micrometers wide. Once the cells become adherent to the top of the walls, they are forced to explore the vertical surfaces to discover “how to survive” and proliferate; so, the cells begin to stretch the cytoplasm in the vertical direction, in search of a stable contact point or an anchor. Part of the cell body might, therefore, move in that direction and the cell could survive with part of the body extended into the gap and vertically attached to the wall. When cells are mature for duplication, this step can be done both on the narrow flat region on top of the wall or on the wider vertical surface exploited for some extension. Experimental results show that the ability to perform this sequence of events is strongly dependent on cell type, that must show the ability to stretch parts of his body to find points of contact and must be able to survive and proliferate in a vertically confined space. As a result of repeated culture experiments involving a significant number of cell lines, we can state that this feature is typical of cell lines that show a mesenchymal behavior.

We also studied the effect of the wall length, thus rigidity, on the cell capability to colonize in depth the HAR PhCs. Cultures were repeated with MRC-5V1 and HT1080 cells on devices with shorter walls (400 µm), and a fluorescence image relative to the HT1080 cells is shown in Figure 5b. These findings demonstrate a similar behavior of cells on PhC with long walls (Figure 5a).

We finally investigated if the colonization of the deep gaps in the HAR PhCs might be influenced by the spontaneous cell sedimentation into the silicon grooves. Figure 6 shows the comparison among the results of a standard incubation (Figure 6a), and of deposition experiments at room temperature (Figure 6b) and at 37°C (Figure 6c). These data refer to HT1080 cells on PhC structures. In Figure 6a, the fluorescence image refers to a standard incubation at 37°C, and clearly shows cells inside the gaps. The clearly visible “fluorescent red bars” come from stretched nuclei deeply located inside the silicon grooves, as also explained in Figure S1. Figure 6b, relative to a cell deposition at room temperature, shows that most of the cells remain on top of the walls, maintaining their round-shaped nuclei; occasionally, cells seem to penetrate into the gaps but without the evidence to fill the available space. In Figure 6c, relative to cell deposition at 37°C, cells are arranged as a sheet on top of the walls and the nuclear DNA looks like split between adjacent gaps. Thus, the cell morphology is similar to the one obtained at room temperature with a slight segmentation effect enhanced by the temperature increase. The reduced medium viscosity, due to the higher temperature, as well as the capillary forces induce a pulling effect of the cell nuclei into the gap. These results demonstrate that an active proliferative process is required to obtain a regular cell growth on the vertical walls inside the deep gaps of a PhC.

## Discussion

We have carried out investigations on the growth behavior of different cell models on three-dimensional silicon devices containing vertical, high aspect-ratio photonic crystals. These results support our findings that only cells prevalently expressing a mesenchymal phenotype are able to extend their cytoplasm, through an active process, for growing inside the gaps. Meanwhile, epithelial cells tend mainly to grow and form colonies on the top of the silicon walls. Nevertheless, the behavior of all tested cell lines is similar on glass slides as well as on flat silicon. Silicon dice incorporating HAR PhCs can be thus identified as 3-D microincubators with a phenotype-selective capability.

Demonstration of the possibility to incorporate a cell-model in a photonic crystal optical transducer represents the initial step toward the development of a label-free cell-based biosensor suitable to be turned into a lab-on-chip, capable of responding to a wide range of biologically active compounds. Our results could find application in a variety of experimental settings characterized by changes in cell morphology, such as apoptosis and EMT, which play a relevant role in drug discovery as well as on cancer progression and tumor metastasis [36] and, more in general, for the elaboration of innovative diagnostic and therapeutic strategies.

Aims of future research work will be design, fabrication and testing of a lab-on-chip, incorporating the selective 3-D microincubator, to be used for various experiments, with additional advantages in terms of cell and reagents consumption.

## Supporting Information

Figure S1 Fluorescence images to highlight how we can distinguish cells inside the gaps from cells on top of the walls. The same region of a PhC is reported in both photos. a: Simultaneous dual color (green-FITC and red-PI) imaging. b: Single color (red-PI) imaging. Rounded nuclei correspond to cells on top of the walls, elongated nuclei are typical of cells inside the gaps.
(TIF)
