# Correction: A New Cell-Selective Three-Dimensional Microincubator Based on Silicon Photonic Crystals

**DOI:** 10.1371/annotation/30b05d2d-7b79-458b-88f7-5d06aaab95b7

**Published:** 2012-11-21

**Authors:** Francesca Carpignano, Gloria Silva, Salvatore Surdo, Valentina Leva, Alessandra Montecucco, Francesca Aredia, Anna Ivana Scovassi, Sabina Merlo, Giuseppe Barillaro, Giuliano Mazzini

There were errors in the Funding section. The correct funding information is as follows: This work was partially supported by Fondazione Alma Mater Ticinensis, Pavia, Italy, and by Fondazione Cariplo, Milan, Italy, grant no. 2011-0308. V. Leva was supported by a postdoctoral fellowship from Fondazione Adriano Buzzati Traverso, Italy. F. Aredia was supported by Regione Lombardia, Italy (Project Plant Cell). The funders had no role in study design, data collection and analysis, decision to publish, or preparation of the manuscript. 

