# Supplementary figures and images for: A New Cell-Selective Three-Dimensional Microincubator Based on Silicon Photonic Crystals
Source: PLoS One. 2012 Nov 6;7(11):e48556. doi: 10.1371/journal.pone.0048556 (PMC3490954; doi:10.1371/journal.pone.0048556)

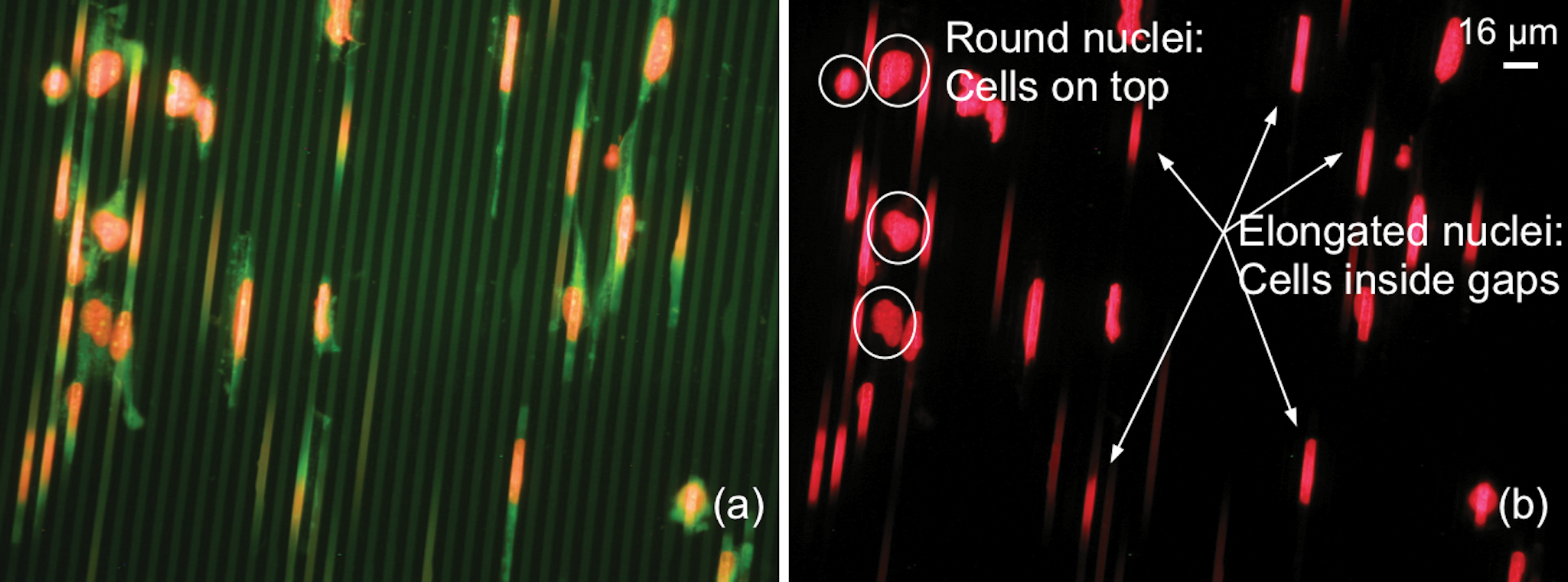

Supplement: Figure S1 — Fluorescence images to highlight how we can distinguish cells inside the gaps from cells on top of the walls. The same region of a PhC is reported in both photos. a: Simultaneous dual color (green-FITC and red-PI) imaging. b: Single color (red-PI) imaging. Rounded nuclei correspond to cells on top of the walls, elongated nuclei are typical of cells inside the gaps. (TIF) [file pone.0048556.s001.tif]
